# Molecular Typing of Mycobacterium Tuberculosis Complex by 24-Locus Based MIRU-VNTR Typing in Conjunction with Spoligotyping to Assess Genetic Diversity of Strains Circulating in Morocco

**DOI:** 10.1371/journal.pone.0135695

**Published:** 2015-08-18

**Authors:** Nada Bouklata, Philip Supply, Sanae Jaouhari, Reda Charof, Fouad Seghrouchni, Khalid Sadki, Youness El Achhab, Chakib Nejjari, Abdelkarim Filali-Maltouf, Ouafae Lahlou, Rajae El Aouad

**Affiliations:** 1 National Tuberculosis Reference Laboratory, National Institute of Hygiene, Rabat, Morocco; 2 INSERMU1018, Lille, France; 3 CNRS UMR8204, Lille, France; 4 Center for Infection and Immunity of Lille (CIIL), Institut Pasteur de Lille, France; 5 Université de Lille, Lille, France; 6 Laboratory of Microbiology and Molecular Biology, Faculty of Sciences, University Mohammed V, Rabat, Morocco; 7 Department of Epidemiology and Public Health, Faculty of Medicine and Pharmacy of Fes, Sidi Mohamed Ben Abdillah University, Fes, Morocco; 8 School of Public Health and Management System Health, University Mohamed VI of Sciences and Health, Casablanca, Morocco; 9 Genoscreen, Lille, France; 10 Laboratory of Cell Immunology, Department of Immunology, National Institute of Hygiene, Rabat, Morocco; St. Petersburg Pasteur Institute, RUSSIAN FEDERATION

## Abstract

**Background:**

Standard 24-locus Mycobacterial Interspersed Repetitive Unit Variable Number Tandem Repeat (MIRU-VNTR) typing allows to get an improved resolution power for tracing TB transmission and predicting different strain (sub) lineages in a community.

**Methodology:**

During 2010–2012, a total of 168 Mycobacterium tuberculosis Complex (MTBC) isolates were collected by cluster sampling from 10 different Moroccan cities, and centralized by the National Reference Laboratory of Tuberculosis over the study period. All isolates were genotyped using spoligotyping, and a subset of 75 was genotyped using 24-locus based MIRU-VNTR typing, followed by first line drug susceptibility testing. Corresponding strain lineages were predicted using MIRU-VNTR*plus* database.

**Principal Findings:**

Spoligotyping resulted in 137 isolates in 18 clusters (2–50 isolates per cluster: clustering rate of 81.54%) corresponding to a SIT number in the SITVIT database, while 31(18.45%) patterns were unique of which 10 were labelled as “unknown” according to the same database. The most prevalent spoligotype family was LAM; (n = 81 or 48.24% of isolates, dominated by SIT42, n = 49), followed by Haarlem (23.80%), T superfamily (15.47%), >Beijing (2.97%), > U clade (2.38%) and S clade (1.19%). Subsequent 24-Locus MIRU-VNTR typing identified 64 unique types and 11 isolates in 5 clusters (2 to 3isolates per cluster), substantially reducing clusters defined by spoligotyping only. The single cluster of three isolates corresponded to two previously treated MDR-TB cases and one new MDR-TB case known to be contact a same index case and belonging to a same family, albeit residing in 3 different administrative regions. MIRU-VNTR loci 4052, 802, 2996, 2163b, 3690, 1955, 424, 2531, 2401 and 960 were highly discriminative in our setting (HGDI >0.6).

**Conclusions:**

24-locus MIRU-VNTR typing can substantially improve the resolution of large clusters initially defined by spoligotyping alone and predominating in Morocco, and could therefore be used to better study tuberculosis transmission in a population-based, multi-year sample context.

## Introduction

Despite the existence of effective antituberculosis drugs, tuberculosis (TB) continues to be a major global health challenge with an estimated 9 million new active cases and 1.5 million TB deaths annually (just under 1 million). Moreover, control efforts to fight TB are threatened by the emergence of different forms of drug resistance [1,2]. In Morocco, TB is a major public health problem with a relatively high incidence reaching 83 new cases for 100000 inhabitants [3]. TB affects especially young adults and therefore has a high impact on the socio-economic situation of the country. TB control remains a priority in Morocco, thus a better understanding of TB transmission could help to identify risk settings as well as to improve contact tracing.

Molecular typing of MTBC is a powerful adjunct to TB control e.g, to monitor the disease transmission, and to detect or confirm outbreaks. In the last decade, the optimized 15 to 24-locus MIRU-VNTR typing system has been proposed for international standardization [4]. This PCR-based system, optionally combined with spoligotyping [5], has been shown to provide a similar resolution power relative to the previous IS*6110* restriction fragment length polymorphism (RFLP) standard for the study of TB transmission in different Western European settings [6–8]. Moreover, MIRU-VNTR typing is also useful for studying at relatively high resolution the diversity and clonal expansion of particular strain or lineages [9–11].

Only a few studies have investigated MTBC genetic diversity in Morocco [12–17]. El Baghdadi et *al*.(1997) and Diraa et *al*.(2005), used IS*6110*-based Restriction Fragment Length Polymorphism (RFLP), whereas Tazi et *al*.(2004,2007), Lahlou et *al*.(2012) and Chaoui et *al*. (2014) used spoligotyping alone or in combination with a 12-locus MIRU-VNTR typing for molecular epidemiological analyses of Moroccan isolates [13,15–17]. The purpose of the present study was therefore to assess standard 24-locus based MIRU-VNTR typing for the first time, on a panel of MTBC isolates collected from diverse geographical cities from Morocco. We tested 168 isolates by spoligotyping, then we evaluated the usefulness of this standardized 24-locus based MIRU-VNTR typing on a subset of 75 MTBC. The specific aims of this study were to evaluate the diversity of circulating MTBC strains at a higher resolution compared to most previous studies and to establish possible links between drug resistance profiles and molecular types.

## Material and Methods

### Study population and setting

TB patients from eight regions of different ethnic groups of Morocco were included in the study. MTBC strains were collected within the framework of immuno-genetics study of the tuberculosis in the population over a period of 3 years; between January 2010 and December 2012. For this molecular epidemiological study, patients were recruited from 12 public health centers and 2 university hospitals. Pulmonary samples were collected from 10 Centers of TB Treatment and Respiratory Disease (CTRD) located in Marrakech, Tanger, Oujda, Fes, Meknes, Sidi Kacem, Sale, Temara, Casablanca and Rabat, whereas extrapulmonary samples (pleural fluid and gastric liquid) were collected from two university hospitals in Casablanca and Rabat. These cities are known to be hot spot areas of TB at National level.

#### Patient isolates

A total of 471 clinical samples were recruited in this study corresponding to 167 patients, from which 155 (92.81%) had pulmonary TB (sputum, sputum induced by fibro-optic bronchoscopy, bronchial wash and bronchial aspirations), 11 (6.58%) patients had extrapulmonary TB (pleural fluid and gastric liquid) and 1 (0.59%) patient with both (pulmonary and extrapulmonary TB). Two patients were re-treatment cases, while the remaining 165 were new cases (Table 1). All pulmonary patients had sputum microscopy positive according to their clinical manifestation; pulmonary and extrapulmonary TB cases were included by cluster sampling from 10 cities (Rabat, Temara, Sale, Casablanca, Marrakech, Sidi Kacem, Meknes, Fes, Oujda and Tanger). Sputum smear microscopy and culture for the collected samples were performed in regional laboratories, then the isolates were submitted to National Reference Laboratory of Tuberculosis (LNRT) at the National Institute of Hygiene in Rabat, for identification and drug susceptibility testing (DST) to first line drugs. Culture of pleural liquid (PL) samples was performed at LNRT.

**Table 1:** Demographic and epidemiological data of the studied population from Morocco (n = 167)

Administrative regions	Isolation city	n(%)	Sex-ratio (M/F)	Mean age (Ecartype) (Year)	New cases n(%)	Previously treated cases n(%)
Grand Casablanca	Casa	5(2.98)	4/1	44.8(25.74)	5(100)	0
Fes-Boulmane	Fes	16(9.53)	12/4	29.56(7.6)	16(100)	0
Marrakech-Tansift- Al Haouz	Marrakech	34(20.23)	27/7	34.17(10.35)	34(100)	0
Region Oriental	Oujda	4(2.38)	2/2	39.25(3.20)	4(100)	0
Rabat-Sale-Zemmour-Zaêr	Rabat	66(39.52)	50/16	33.92(13.32)	66(100)	0
	Sale	16(9.52)	10/6	26.25(7.39)	16(100)	0
	Temara	4(2.38)	100% M	41,5(26.73)	4(100)	0
Gharb-Chrarda-BniHssen	Sidi Kacem	1(0.6)	^d^NA	^d^NA	1(100)	0
Meknes-Tafilalt	Meknes	2(1.19)	100% F	23(4.24)	0	2(100)
Tanger-Tetouan	Tanger	19(11.3)	14/5	32.68(9.51)	19(100%)	0
Total	10	167	124/43	32.25(12.23)	165	2

M: Male; F: Female

^d^NA: Not applicable since only one patient originated from this city.

Ethical approval statement was obtained (Reference number 1169) from ethics committee named “comité d’éthique de Médicine et de Pharmacie de Rabat”; participants provide their written informed consent and the ethics committee approves these consent procedures and the present study.

All TB patients were diagnosed according to the national guidelines in Morocco.

### Strains isolation and drug susceptibility testing

The clinical samples were cultured on Lowenstein-Jensen (L/J) culture media and on Growth Indicator Tubes (MGIT) 960 culture tubes inoculated in Bactec System. The isolates (n = 168) were subjected to identification as MTBC using biochemical tests including production of Niacin (Strip Niacin [Becton Dickinson, CA, USA]), catalase activity and susceptibility testing of MTBC to P-Nitrobenzoic acid (PNB), Thiophene-2-carboxylic acid hydrazide (TCH) and paraaminosalicylic acid (PAS) [18]. First line drug susceptibility testing was performed using the 1% proportion method for isoniazid (INH), rifampicin (RMP), streptomycin (SM) and ethambutol (EMB) at the following concentrations: 0.2 mg/ml, 40 mg/ml, 4 mg/ml and 2 mg/ml respectively on L/J medium [19] and using BACTEC MGIT 960 SIRE kit [Becton Dickinson, CA, USA]. Results were categorized into three major groups, i.e. resistance to a single drug (mono resistance), to more than one drug but not INH and RMP (polyresistance), and resistance to at least both INH and RMP (MDR-TB, [18]). Epidemiological and demographic data such as age, sex, city of birth, address at the time of diagnosis, place of residence and clinical characteristics of the disease were prospectively collected using a questionnaire established within the study framework.

### Molecular methods

Genomic DNA was prepared from scarped colonies in 200 μl of 1xTE buffer (10mM Tris-HCl pH: 8.0, 1mM EDTA pH: 8.0) followed by heat inactivation at 85° for 30 min [20]. Spoligotyping was carried out for 168 isolates, using the commercially available membranes (Ocimum Biosolutions, Hyderabad, India) [5]. Standard 24-locus based MIRU-VNTR typing was performed for 75 isolates using the available commercial kit (Genoscreen, Lille, France) and 16 capillary ABI 3730 genetic analyzer (Applied Biosystems, CA, 78 USA) as described previously [4]. MIRU-VNTR alleles were determined by using Genemapper V-4.0 (Applied Biosystems, CA,79 USA) and data were compiled by using MIRU-VNTR Data Manager software (Genoscreen, Lille, France).

### Molecular data analysis

Spoligotyping results were converted into octal codes and entered in SITVIT database [21] for analysis. Major spoligotypes families were assigned according to signatures provided in the database. Distinction between evolutionary, ancient, and modern lineages of tubercle bacilli was made as described [22–27]. MTBC genetic lineages were predicted using online tools available from MIRU-VNTR*plus* website (www.miruvntrplus.org [28]) according to the previously described strategy combining best-match and phylogenetic based analysis [29] and by using information in the SITVIT database (fr:8081/SITVIT ONLINE/indexjsp). Molecular clustering of the isolates was determined by constructing a dendogram based on spoligotyping and MIRU-VNTR data. A strain cluster was defined as two or more isolates sharing completely identical fingerprints based on both methods. Discriminatory power of a typing method (or a combination of methods) was calculated using the Hunter and Gaston Discriminatory Index (HGDI) [30]. Cases of isolates displaying double alleles in one locus suggestive of clonal microevolution [31], or in two or more VNTR loci suggestive of mixed genotypes, which could reflect either mixed infection or contamination [32] were retested.

## Results

### Study Population

A total of 168 isolates were enrolled in this study, from 167 patients were selected from 10 cities located in 8 of 16 administrative regions of Morocco (51.79% from Rabat-Sale-Zemmour-Zaêr, 20.23% from Marrakech-Tansift-Al Haouz, 11.3% from Tanger-Tetouan, 9.53% from Fes-Boulmane, 2.98% from Grand Casablanca, 2.38% from Region Oriental, 1.19% from Meknes-Tafilalt and 0.6% from Gharb-Chrarda-BniHssen). These cities are known to be hot spot areas of TB. The demographic and epidemiological data summarized in Table 1 showed that the age of patients ranged from 18 to 81 years (mean 49.5 years). Gender information was available for all cases: males represented 74.25% of the cases, with a male to female sex-ratio of 2.9 (124/43). There was no correlation between localities (administrative regions) and age/sex-ratio of patients. Clinical status was available for all TB patients: 98.8% (165/167) were new cases and 1.19% (2/167) were previously treated. 92.81% (155/167) had pulmonary TB, while 6.58% (11/167) had extrapulmonary TB. One patient (0.59%) had both pulmonary and extrapulmonary TB (Table 1).

### Drug susceptibility

The drug susceptibility testing (DST) data was available for all cases, and showed that 88.7% (149/168) of the strains were pansusceptible, while the remaining 11.3% (19/168) showed resistance to one or more drugs. Among the resistant strains, monoresistance to INH, SM and EMB was found in 6.0%, 0.6% and 0.6% of the tested strains respectively, as opposed to none for RMP. Of note, MDR isolates represented only 5 (3.0%) of 165 new TB cases included in the study. In contrast, the two remaining retreatment cases were MDR.

### Spoligotyping data analysis

Analysis of the spoligotyping data was done by assigning shared international type (SIT) numbers and genotypic spoligotype family designations in comparison with SpolDB4 [21] and its updated SITVIT version [22] (S1 Table). The global sample containing 10/168 (6.0%) isolates that could not be assigned into well-determined designations in both, and as such were labeled as ‘‘unknown”. In contrast, the remaining, 158 isolates belonged to 39 classified SITs in the international SITVIT database. A total of 137/168 (81.54%) clinical isolates were clustered 18 clusters containing 2–50 isolates per cluster while 31 were unique (21 with an assigned SIT, and 10 unknown from SITVIT).

The major spoligotype families observed ranked as follows: Latin-American & Mediterranean or LAM, 81/168 (48.21%) with the following subfamily distribution: LAM3 n = 8; LAM5 n = 1; LAM6 n = 1; LAM9 n = 67; CAM alias Cameroon, n = 4; Haarlem, 40/168 (23.80%) with the following subfamily distribution H1 n = 11; H3 n = 29; T superfamily 26/168 (15.47%) with the following distribution: T1 n = 24; T2 n = 2; Beijing, 5/168 (2.97%); U clade, 4/168 (2.38%) and S clade, 2/168 (1.19%). No X lineage strains or EAI (East-African Indian) strains were found in the present investigation.

Regarding the frequencies of the major shared types, SIT42 (LAM9) strongly predominated in our setting with 49/168 (29.16%) of the isolates. It was followed by SIT53 (T spoligotype group) with 19/168 isolates (11.3%) and SIT50 (Haarlem) with 17/168 isolates (10.1%). These three predominant SITs were widespread in all Moroccan cities included in this study. Interestingly, 8 isolates, with a priori more unexpected spoligotypes, corresponding to SIT61 (CAM: n = 4), to SIT1070 (U: n = 3) and SIT443 (U: n = 1), were isolated either in the capital city’s administrative region of Rabat-Sale-Zemmour-Zaer, or in the cosmopolite and touristic administrative regions of Marrakech-Tensift-Al Haouz and Fes-Boulmane (Table 2). Likewise, we also found 5 isolates (2.97%) with a SIT1 (Beijing) spoligotype among cases from Marrakech-Tansift-Al Haouz, Rabat-Sale-Zemmour-Zaer and Grand Casablanca capital of touristic region.

**Table 2:** Detailed Results obtained including demographic, drug-resistance and genotyping information on 5 Clusters and 69 unique patterns defined by identical spoligotyping and 24-loci MIRU from 75 *M*.*tuberculosis* strain isolated in Morocco.

ID	Year of Isolation	Sex	Age	City	site of Infection	Spoligotype Description	Cluster(SIT/Clade)	MIRU24 profil	MLVA MtbC15-9	DST status			
83	2011	M	81	Casablanca	Extrapulmonary	▫▫▫▫▫▫▫▫▫▫▫▫▫▫▫▫▫▫▫▫▫▫▫▫▫▫▫▫▫▫▫▫▫▫▪▪▪▪▪▪▪▪▪	NC(1/Beijing)	244233352644425173353723	100–32	r	s	s	r
18	2011	M	26	Rabat	Pulmonary	▪▪▪▪▪▪▪▪▪▪▪▪▪▪▪▪▪▪▪▪▪▪▪▪▪▫▫▫▫▫▫▪▫▫▫▫▪▪▪▪▪▪▪	Cluster A(47/H1)	223235332332423153333732	322–31	s	s	s	s
27	2011	M	43	Rabat	Pulmonary	▪▪▪▪▪▪▪▪▪▪▪▪▪▪▪▪▪▪▪▪▪▪▪▪▪▫▫▫▫▫▫▪▫▫▫▫▪▪▪▪▪▪▪	Cluster A(47/H1)	223235332432423153323732	1509–31	r	s	s	s
32	2011	M	32	Fes	Pulmonary	▪▪▪▪▪▪▪▪▪▪▪▪▪▪▪▪▪▪▪▪▪▪▪▪▪▫▫▫▫▫▫▪▫▫▫▫▪▪▪▪▪▪▪	Cluster A(47/H1)	223335332432423153334832	Unk-31	s	s	s	s
77	2011	M	26	Sale	Pulmonary	▪▪▪▪▪▪▪▪▪▪▪▪▪▪▪▪▪▪▪▪▪▪▪▪▪▫▫▫▫▫▫▪▫▫▫▫▪▪▪▪▪▪▪	Cluster A(47/H1)	223215372532423133334732	Unk-31	s	s	s	s
11	2011	M	31	Rabat	Pulmonary	▪▪▪▪▪▪▪▪▪▪▪▪▪▪▪▪▪▪▪▪▪▪▪▪▪▪▪▪▪▪▫▪▫▫▫▫▪▪▪▪▪▪▪	Cluster B(50/H3)	223225321632423153333622	307–76	s	s	s	s
17	2011	M	19	Rabat	Pulmonary	▪▪▪▪▪▪▪▪▪▪▪▪▪▪▪▪▪▪▪▪▪▪▪▪▪▪▪▪▪▪▫▪▫▫▫▫▪▪▪▪▪▪▪	Cluster B(50/H3)	233235322634425154233432	9927–415	s	s	s	s
20	2012	M	28	Rabat	Pulmonary	▪▪▪▪▪▪▪▪▪▪▪▪▪▪▪▪▪▪▪▪▪▪▪▪▪▪▪▪▪▪▫▪▫▫▫▫▪▪▪▪▪▪▪	Cluster B(50/H3)	223232372532423123333232	Unk-31	r	s	s	r
26	2011	M	20	Sale	Extrapulmonary	▪▪▪▪▪▪▪▪▪▪▪▪▪▪▪▪▪▪▪▪▪▪▪▪▪▪▪▪▪▪▫▪▫▫▫▫▪▪▪▪▪▪▪	Cluster B(50/H3)	134243352324126153332932	358–51	s	s	s	s
44	2011	F	26	Fes	Pulmonary	▪▪▪▪▪▪▪▪▪▪▪▪▪▪▪▪▪▪▪▪▪▪▪▪▪▪▪▪▪▪▫▪▫▫▫▫▪▪▪▪▪▪▪	Cluster B(50/H3)	223235321332425153333622	Unk-116	s	r	s	s
63	2011	M	29	Rabat	Pulmonary	▪▪▪▪▪▪▪▪▪▪▪▪▪▪▪▪▪▪▪▪▪▪▪▪▪▪▪▪▪▪▫▪▫▫▫▫▪▪▪▪▪▪▪	Cluster B(50/H3)	233235322634425154233232	Unk-415	s	s	s	s
65	2011	M	30	Temara	Pulmonary	▪▪▪▪▪▪▪▪▪▪▪▪▪▪▪▪▪▪▪▪▪▪▪▪▪▪▪▪▪▪▫▪▫▫▫▫▪▪▪▪▪▪▪	Cluster B(50/H3)	123236372434425143333632	Unk-52	s	s	s	s
70	2011	F	25	Rabat	Pulmonary	▪▪▪▪▪▪▪▪▪▪▪▪▪▪▪▪▪▪▪▪▪▪▪▪▪▪▪▪▪▪▫▪▫▫▫▫▪▪▪▪▪▪▪	Cluster B(50/H3)	123233332434425153333632	Unk-52	r	s	s	s
79	2012	M	31	Rabat	Pulmonary	▪▪▪▪▪▪▪▪▪▪▪▪▪▪▪▪▪▪▪▪▪▪▪▪▪▪▪▪▪▪▫▪▫▫▫▫▪▪▪▪▪▪▪	Cluster B(50/H3)	233235322634425154233432	9927–415	s	s	s	s
75	2011	M	37	Rabat	Pulmonary	▪▪▪▪▪▪▪▪▪▪▪▪▪▪▪▪▪▪▪▪▪▪▪▪▪▪▪▪▪▪▫▪▫▫▫▫▪▪▪▪▪▪▪	Cluster B(50/H3)	123236332434425153333632	2433–52	s	s	s	s
21	2011	M	40	Fes	Pulmonary	▪▪▪▪▪▪▪▪▪▪▪▪▪▪▪▪▪▪▪▪▪▪▫▪▪▪▪▪▪▪▫▪▫▫▫▫▪▪▪▪▪▪▪	Cluster C(741/H3)	223235332334425153333432	1808–15	s	s	s	s
47	2011	M	26	Fes	Pulmonary	▪▪▪▪▪▪▪▪▪▪▪▪▪▪▪▪▪▪▪▪▪▪▫▪▪▪▪▪▪▪▫▪▫▫▫▫▪▪▪▪▪▪▪	Cluster C(741/H3)	223215332434425153333732	1480–15	s	s	s	s
55	2011	M	29	Rabat	Pulmonary	▪▪▪▪▪▫▪▪▪▪▪▪▪▪▪▪▪▪▪▪▪▪▪▪▪▪▪▪▪▪▫▪▫▫▫▫▪▪▫▫▪▪▪	NC(1743/H3)	223235271332423153333722	Unk-76	s	s	s	s
1	2010	F	43	Marrakech	Pulmonary	▪▪▪▪▪▪▪▪▪▪▪▪▪▪▪▪▪▪▪▪▪▪▫▫▫▪▪▪▪▪▪▪▫▫▫▫▪▪▪▪▪▪▪	Cluster D(61/CAM)	223235372332425153333222	Unk-111	s	s	s	s
49	2011	M	34	Fes	Pulmonary	▪▪▪▪▪▪▪▪▪▪▪▪▪▪▪▪▪▪▪▪▪▪▫▫▫▪▪▪▪▪▪▪▫▫▫▫▪▪▪▪▪▪▪	Cluster D(61/CAM)	2242133316442251533311422	Unk-26	s	s	s	s
3	2011	F	42	Oujda	Pulmonary	▪▪▪▪▪▪▪▪▫▫▫▪▫▪▪▪▪▪▪▪▫▫▫▫▪▪▪▪▪▪▪▪▫▫▫▫▪▪▪▫▪▪▪	NC(33/LAM3)	224244322322225162342522	Unk-110	s	s	s	s
69	2011	M	25	Rabat	Pulmonary	▪▪▪▪▪▪▪▪▪▪▪▪▫▪▪▪▪▪▪▪▫▫▫▫▪▪▪▪▪▪▪▪▫▫▫▫▪▪▪▪▪▪▪	NC(93/LAM5)	134264332224127143332832	Unk-68	s	s	s	s
4	2011	M	42	Oujda	Pulmonary	▪▪▪▪▪▪▪▪▪▪▪▪▪▪▪▪▪▪▪▪▫▫▫▫▪▪▪▪▪▪▪▪▫▫▫▫▪▪▪▪▪▪▪	Cluster F(42/LAM9	224213332322126133332022	Unk-259	s	s	s	s
8	2011	M	53	Rabat	Pulmonary	▪▪▪▪▪▪▪▪▪▪▪▪▪▪▪▪▪▪▪▪▫▫▫▫▪▪▪▪▪▪▪▪▫▫▫▫▪▪▪▪▪▪▪	Cluster F(42/LAM9	2420s0435232426123345132	Unk-62	s	s	s	s
10	2011	M	34	Rabat	Pulmonary	▪▪▪▪▪▪▪▪▪▪▪▪▪▪▪▪▪▪▪▪▫▫▫▫▪▪▪▪▪▪▪▪▫▫▫▫▪▪▪▪▪▪▪	Cluster F(42/LAM9	224244322324226173345522	369–62	s	s	s	s
12	2011	M	24	Rabat	Pulmonary	▪▪▪▪▪▪▪▪▪▪▪▪▪▪▪▪▪▪▪▪▫▫▫▫▪▪▪▪▪▪▪▪▫▫▫▫▪▪▪▪▪▪▪	Cluster F(42/LAM9	214222321324242173345222	Unk-Unk	s	s	s	s
16	2011	M	31	Rabat	Extrapulmonary	▪▪▪▪▪▪▪▪▪▪▪▪▪▪▪▪▪▪▪▪▫▫▫▫▪▪▪▪▪▪▪▪▫▫▫▫▪▪▪▪▪▪▪	Cluster F(42/LAM9	1342243352324126153332932	358–51	s	s	s	s
24	2011	F	22	Casablkanca	Extrapulmonary	▪▪▪▪▪▪▪▪▪▪▪▪▪▪▪▪▪▪▪▪▫▫▫▫▪▪▪▪▪▪▪▪▫▫▫▫▪▪▪▪▪▪▪	Cluster F(42/LAM9	134234372224126133332732	Unk-51	s	s	s	s
29	2011	F	20	Fes	Pulmonary	▪▪▪▪▪▪▪▪▪▪▪▪▪▪▪▪▪▪▪▪▫▫▫▫▪▪▪▪▪▪▪▪▫▫▫▫▪▪▪▪▪▪▪	Cluster F(42/LAM9	1322643322241261633332632	Unk-51	r	s	s	s
30	2010	M	34	Marrakech	Pulmonary	▪▪▪▪▪▪▪▪▪▪▪▪▪▪▪▪▪▪▪▪▫▫▫▫▪▪▪▪▪▪▪▪▫▫▫▫▪▪▪▪▪▪▪	Cluster F(42/LAM9	244244322324224143333522	Unk-82	r	s	s	s
38	2011	M	36	Rabat	Pulmonary	▪▪▪▪▪▪▪▪▪▪▪▪▪▪▪▪▪▪▪▪▫▫▫▫▪▪▪▪▪▪▪▪▫▫▫▫▪▪▪▪▪▪▪	Cluster F(42/LAM9	144254232224126153332732	Unk-51	s	s	s	s
41	2011	M	40	Sale	Pulmonary	▪▪▪▪▪▪▪▪▪▪▪▪▪▪▪▪▪▪▪▪▫▫▫▫▪▪▪▪▪▪▪▪▫▫▫▫▪▪▪▪▪▪▪	Cluster F(42/LAM9	134254212224124153332732	Unk-83	s	s	s	s
43	2011	F	19	Sale	Pulmonary	▪▪▪▪▪▪▪▪▪▪▪▪▪▪▪▪▪▪▪▪▫▫▫▫▪▪▪▪▪▪▪▪▫▫▫▫▪▪▪▪▪▪▪	Cluster F(42/LAM9	134244332224126153332532	239–51	s	s	s	s
45	2011	F	26	Fes	Pulmonary	▪▪▪▪▪▪▪▪▪▪▪▪▪▪▪▪▪▪▪▪▫▫▫▫▪▪▪▪▪▪▪▪▫▫▫▫▪▪▪▪▪▪▪	Cluster F(42/LAM9	145244322324225182346522	Unk-72	s	s	s	**s**
48	2011	M	20	Fes	Pulmonary	▪▪▪▪▪▪▪▪▪▪▪▪▪▪▪▪▪▪▪▪▫▫▫▫▪▪▪▪▪▪▪▪▫▫▫▫▪▪▪▪▪▪▪	Cluster F(42/LAM9	135274332224126153332732	Unk-51	s	s	s	s
54	2011	F	42	Tanger	Pulmonary	▪▪▪▪▪▪▪▪▪▪▪▪▪▪▪▪▪▪▪▪▫▫▫▫▪▪▪▪▪▪▪▪▫▫▫▫▪▪▪▪▪▪▪	Cluster F(42/LAM9	2442234322424226133131722	300–53	s	s	s	s
57	2011	M	42	Tanger	Pulmonary	▪▪▪▪▪▪▪▪▪▪▪▪▪▪▪▪▪▪▪▪▫▫▫▫▪▪▪▪▪▪▪▪▫▫▫▫▪▪▪▪▪▪▪	Cluster F(42/LAM9	134224332224126143332732	9823–51	s	s	s	s
58	2011	M	24	Sale	Pulmonary	▪▪▪▪▪▪▪▪▪▪▪▪▪▪▪▪▪▪▪▪▫▫▫▫▪▪▪▪▪▪▪▪▫▫▫▫▪▪▪▪▪▪▪	Cluster F(42/LAM9	2432443323344251533431032	Unk-15	s	s	s	s
59	2011	F	18	Sale	Pulmonary	▪▪▪▪▪▪▪▪▪▪▪▪▪▪▪▪▪▪▪▪▫▫▫▫▪▪▪▪▪▪▪▪▫▫▫▫▪▪▪▪▪▪▪	Cluster F(42/LAM9	2432443723324251533431032	Unk-111	s	s	s	s
60	2011	F	21	Rabat	Pulmonary	▪▪▪▪▪▪▪▪▪▪▪▪▪▪▪▪▪▪▪▪▫▫▫▫▪▪▪▪▪▪▪▪▫▫▫▫▪▪▪▪▪▪▪	Cluster F(42/LAM9	134224352222126133332332	Unk-1011	s	s	s	s
62	2011	M	34	Sale	Pulmonary	▪▪▪▪▪▪▪▪▪▪▪▪▪▪▪▪▪▪▪▪▫▫▫▫▪▪▪▪▪▪▪▪▫▫▫▫▪▪▪▪▪▪▪	Cluster F(42/LAM9	224222322324226173344322	11247–62	r	s	s	s
64	2011	M	81	Temara	Pulmonary	▪▪▪▪▪▪▪▪▪▪▪▪▪▪▪▪▪▪▪▪▫▫▫▫▪▪▪▪▪▪▪▪▫▫▫▫▪▪▪▪▪▪▪	Cluster F(42/LAM9	244214262324116143332122	Unk-92	s	s	s	S
66	2011	M	20	Sale	Pulmonary	▪▪▪▪▪▪▪▪▪▪▪▪▪▪▪▪▪▪▪▪▫▫▫▫▪▪▪▪▪▪▪▪▫▫▫▫▪▪▪▪▪▪▪	Cluster F(42/LAM9	223122371532423123333622	Unk-76	s	s	s	S
67	2011	M	32	Sale	Pulmonary	▪▪▪▪▪▪▪▪▪▪▪▪▪▪▪▪▪▪▪▪▫▫▫▫▪▪▪▪▪▪▪▪▫▫▫▫▪▪▪▪▪▪▪	Cluster F(42/LAM9	134284332224126153332632	Unk-51	s	s	s	S
68	2012	M	20	Sale	Pulmonary	▪▪▪▪▪▪▪▪▪▪▪▪▪▪▪▪▪▪▪▪▫▫▫▫▪▪▪▪▪▪▪▪▫▫▫▫▪▪▪▪▪▪▪	Cluster F(42/LAM9	244244321322225182345122	Unk-Unk	s	s	s	S
72	2011	M	21	Rabat	Pulmonary	▪▪▪▪▪▪▪▪▪▪▪▪▪▪▪▪▪▪▪▪▫▫▫▫▪▪▪▪▪▪▪▪▫▫▫▫▪▪▪▪▪▪▪	Cluster F(42/LAM9	244244322324226163345522	11304–62	s	s	s	S
94	2011	M	25	Rabat	Pulmonary	▪▪▪▪▪▪▪▪▪▪▪▪▪▪▪▪▪▪▪▪▫▫▫▫▪▪▪▪▪▪▪▪▫▫▫▫▪▪▪▪▪▪▪	Cluster F(42/LAM9	242244322322226163345522	Unk-259	s	s	s	S
166	2012	F	26	Meknes	Pulmonary	▪▪▪▪▪▪▪▪▪▪▪▪▪▪▪▪▪▪▪▪▫▫▫▫▪▪▪▪▪▪▪▪▫▫▫▫▪▪▪▪▪▪▪	Cluster F(42/LAM9	244244321322225172346522	10248-Unk	r	r	s	r
168	2012	F	20	Meknes	Pulmonary	▪▪▪▪▪▪▪▪▪▪▪▪▪▪▪▪▪▪▪▪▫▫▫▫▪▪▪▪▪▪▪▪▫▫▫▫▪▪▪▪▪▪▪	Cluster F(42/LAM9	244244321322225172346522	10248-Unk	r	r	s	r
169	2012	M	19	Sidi Kacem	Pulmonary	▪▪▪▪▪▪▪▪▪▪▪▪▪▪▪▪▪▪▪▪▫▫▫▫▪▪▪▪▪▪▪▪▫▫▫▫▪▪▪▪▪▪▪	Cluster F(42/LAM9	244244321322225172346522	10248-Unk	r	r	s	r
170	2011	M	43	Marrakech	Pulmonary	▪▪▪▪▪▪▪▪▪▪▪▪▪▪▪▪▪▪▪▪▫▫▫▫▪▪▪▪▪▪▪▪▫▫▫▫▪▪▪▪▪▪▪	Cluster F(42/LAM9	244214322232422463332622	220–82	s	s	s	s
19	2011	M	37	Oujda	Pulmonary	▪▪▪▪▫▪▪▪▪▪▪▪▪▪▪▪▪▪▪▪▫▫▫▫▪▪▪▪▪▪▪▪▫▫▫▫▪▪▪▪▪▪▪	Cluster E(822/LAM9)	224213371844225133333222	Unk-26	s	s	s	s
37	2011	M	33	Rabat	Pulmonary	▪▪▪▪▫▪▪▪▪▪▪▪▪▪▪▪▪▪▪▪▫▫▫▫▪▪▪▪▪▪▪▪▫▫▫▫▪▪▪▪▪▪▪	Cluster E(822/LAM9)	23424423222226143333322	Unk-259	r	s	s	s
5	2011	M	27	Marrakech	Pulmonary	▪▪▪▪▪▪▪▪▫▪▪▪▪▪▪▪▪▪▪▪▫▫▫▫▪▪▪▪▪▪▪▪▫▫▫▫▪▪▪▪▪▪▪	NC(1708/LAM9)	224244322324226173347322	271–62	s	s	s	s
7	2011	M	55	Rabat	Pulmonary	▪▪▪▪▪▪▪▪▪▪▪▪▪▪▪▪▪▪▪▪▫▫▫▫▪▪▪▪▪▪▪▪▫▫▫▫▪▪▪▪▪▫▪	NC(1074/LAM9)	244254331324224163333422	Unk-578	s	s	s	s
13	2011	M	60	Rabat	Pulmonary	▪▪▪▪▪▪▪▪▪▪▪▫▫▫▪▪▪▪▪▪▫▫▫▫▪▪▪▪▪▪▪▪▫▫▫▫▪▪▪▪▪▪▪	NC(273/LAM9)	244244322123225192346522	Unk- 488	s	s	s	s
15	2011	F	29	Rabat	Extrapulmonary	▪▪▪▪▪▪▪▪▪▪▫▫▪▪▪▪▪▪▪▪▫▫▫▫▪▪▪▪▪▪▪▪▫▫▫▫▪▪▪▪▪▪▪	NC(252/LAM9)	242244322324226163345522	Unk-62	s	s	s	s
31	2011	M	42	Fes	Pulmonary	▪▪▪▪▪▪▪▪▪▪▪▪▪▪▫▫▫▪▪▪▫▫▫▫▪▪▪▪▪▪▪▪▫▫▫▫▪▪▪▪▪▪▪	NC(731/LAM9)	244214322324226163332622	220–62	s	s	s	s
167	2011	M	24	Fes	Pulmonary	▪▪▪▪▪▪▪▪▪▪▪▪▪▪▪▫▫▪▪▪▫▫▫▫▪▪▪▪▪▪▪▪▫▫▫▫▪▪▪▪▪▪▪	NC(1071/LAM9)	224212332222126163334822	Unk-259	r	r	r	r
2	2011	F	42	Marrakech	Pulmonary	▪▪▪▪▪▪▪▪▪▪▪▪▪▪▪▪▪▪▪▪▪▪▪▪▪▪▪▪▪▪▪▪▫▫▫▫▪▪▪▪▪▪▪	Cluster F(53/T1)	224223422424225143333522	4464–15	s	s	s	s
6	2010	M	52	Rabat	Pulmonary	▪▪▪▪▪▪▪▪▪▪▪▪▪▪▪▪▪▪▪▪▪▪▪▪▪▪▪▪▪▪▪▪▫▫▫▫▪▪▪▪▪▪▪	Cluster F(53/T1)	2421343222224445133346622	Unk-569	s	s	s	s
22	2011	M	40	Fes	Pulmonary	▪▪▪▪▪▪▪▪▪▪▪▪▪▪▪▪▪▪▪▪▪▪▪▪▪▪▪▪▪▪▪▪▫▫▫▫▪▪▪▪▪▪▪	Cluster F(53/T1)	223234332334425153333712	13387–15	s	s	s	s
23	2011	F	31	Fes	Pulmonary	▪▪▪▪▪▪▪▪▪▪▪▪▪▪▪▪▪▪▪▪▪▪▪▪▪▪▪▪▪▪▪▪▫▫▫▫▪▪▪▪▪▪▪	Cluster F(53/T1)	223234332334425153333712	13387–15	s	s	s	s
42	2011	M	27	Sale	Pulmonary	▪▪▪▪▪▪▪▪▪▪▪▪▪▪▪▪▪▪▪▪▪▪▪▪▪▪▪▪▪▪▪▪▫▫▫▫▪▪▪▪▪▪▪	Cluster F(53/T1)	2141251322344225113333832	682–15	s	s	s	s
51	2011	F	36	Oujda	Pulmonary	▪▪▪▪▪▪▪▪▪▪▪▪▪▪▪▪▪▪▪▪▪▪▪▪▪▪▪▪▪▪▪▪▫▫▫▫▪▪▪▪▪▪▪	Cluster F(53/T1)	2432443323344251533431032	Unk-15	s	s	s	s
34	2011	M	32	Rabat	Extrapulmonary	▪▪▪▪▪▪▪▪▪▪▪▪▪▪▪▪▪▪▪▪▪▪▪▪▪▪▪▪▪▪▪▪▫▫▫▫▪▪▪▪▪▪▪	Cluster F(53/T1)	224243322334225153343222	7048–15	s	s	s	s
71	2011	F	42	Sale	Pulmonary	▪▪▪▪▪▪▪▪▪▪▪▪▪▪▪▪▪▪▪▪▪▪▪▪▪▪▪▪▪▪▪▪▫▫▫▫▪▪▪▪▪▪▪	Cluster F(53/T1)	212224131232425113333122	Unk-116	s	s	s	s
9	2011	M	33	Rabat	Pulmonary	▪▪▪▪▪▪▪▪▪▪▪▪▪▪▪▪▪▪▪▪▪▪▪▪▫▫▫▫▫▫▫▫▫▫▫▫▪▪▪▪▪▪▪	NC(602/T1)	223235372532423123333732	Unk-31	r	s	s	s
14	2011	M	33	Rabat	Pulmonary	▪▪▪▪▪▪▪▪▪▪▪▪▪▪▪▪▪▪▪▪▪▪▪▪▪▪▪▪▫▪▪▪▫▫▫▫▪▪▪▪▪▪▪	NC(462/T1)	263244442634425153333612	Unk-15	s	s	s	s
25	2011	M	60	Rabat	Pulmonary	▪▪▪▪▪▪▪▪▫▫▪▪▪▪▪▪▪▪▪▪▪▪▪▪▪▪▪▪▪▪▪▪▫▫▫▫▪▪▪▫▪▪▪	NC(784/T2)	244244322124225192346522	Unk-85	s	s	s	s
73	2011	F	24	Sale	Pulmonary	▪▪▪▪▫▪▪▪▪▪▪▪▪▪▪▪▪▪▪▪▪▪▪▪▪▪▪▫▪▪▫▪▫▫▫▫▪▪▪▪▪▪▪	NC(443/Clade U)	223234331234425153333732	296–26	r	s	s	s
81	2011	F	18	Sale	Pulmonary	▪▪▪▪▪▪▪▪▪▪▪▪▪▪▪▪▪▪▪▪▫▫▫▫▪▪▪▪▪▪▪▪▫▫▫▫▫▪▪▪▪▪▪	NC(1070/Clade U)	134254332224126161332632	Unk-349	s	s	s	s
61	2011	M	28	Rabat	Pulmonary	▫▫▪▪▪▪▪▪▪▪▪▪▪▪▪▪▪▪▪▪▪▪▪▪▪▫▫▫▫▫▫▪▫▫▫▫▪▪▪▪▪▪▪	Unk	223235332432423153333232	2786–31	s	s	s	s
78	2011	F	24	Sale	Pulmonary	▪▪▪▪▪▪▪▫▫▫▫▫▫▪▪▪▪▪▪▪▫▫▫▫▫▫▫▪▪▪▪▪▫▫▫▫▪▪▪▫▫▫▫	Unk	134264332224126133332832	2613–159	s	s	s	s
89	2012	F	18	Tanger	Pulmonary	▪▪▪▪▪▪▪▪▪▪▪▪▪▪▪▪▪▫▫▫▫▫▫▫▫▫▫▫▫▫▫▫▫▫▫▫▫▫▫▫▫▫▫	Unk	134264332224126133332832	446–51	s	s	s	s

ID: Identifying number

DST: Drug SusceptibilityTesting

MLVA-MtbC15-9: Multi Locus Variant Allele-Mycobacterium tuberculosis complex15-9

### Molecular cluster analysis

**Fig 1:**
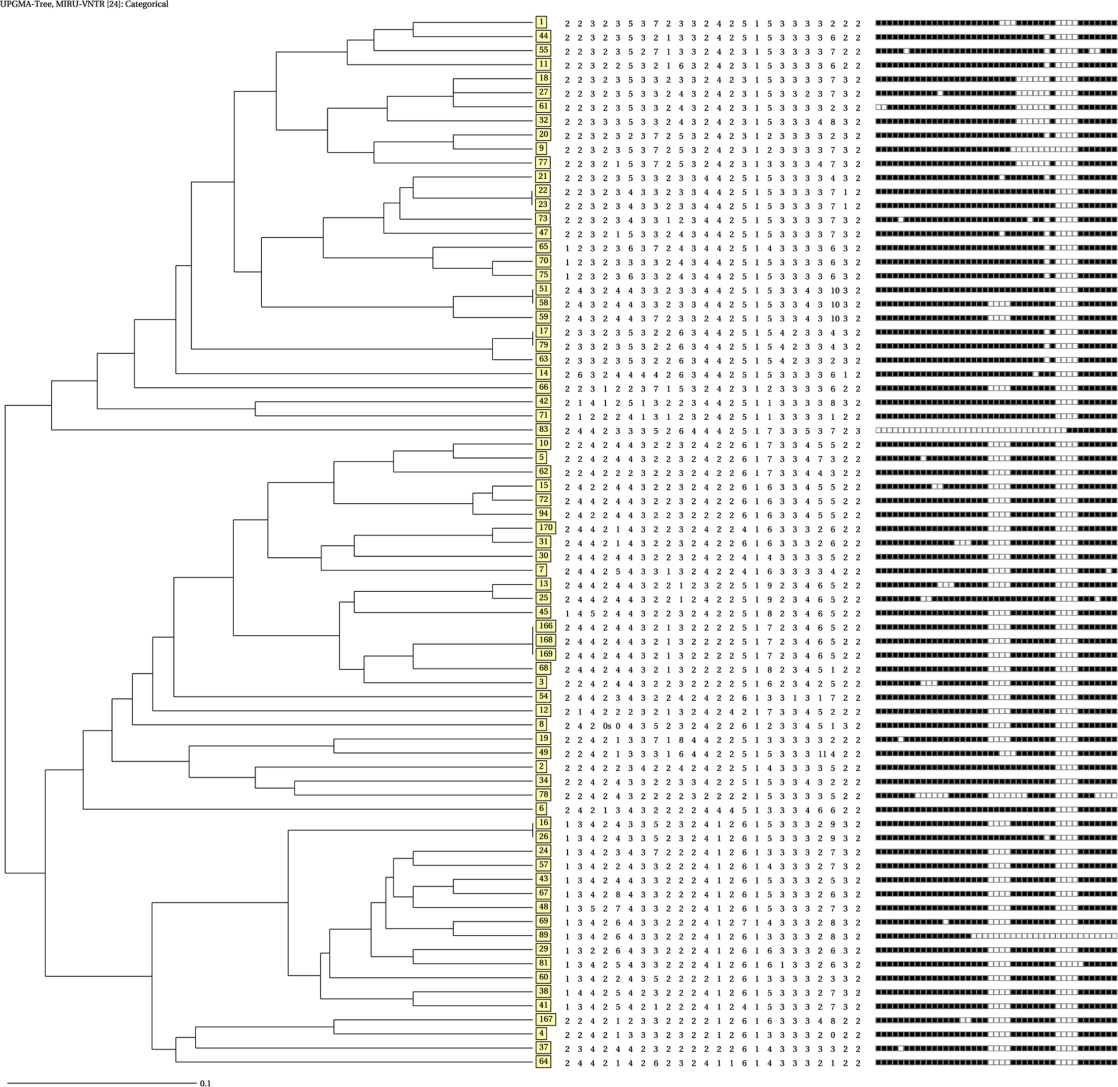
Genetic tree based on spoligotyping and 24-locus MIRU-VNTR data of 75 *M*. *tuberculosis* isolates from10 Moroccan cities. A dendogram was generated using the UPGMA algorithm using tools available from the MIRU-VNTR*plus* identification database (see text). Isolates are identified according to their corresponding spoligotype international type (SIT; boxed), according to the SITVIT database.

A subset of MTBC isolates (75/168) was subjected to 24-locus MIRU-VNTR typing. These isolates were selected to cover the different cities included in the study and different already established spoligotype families; they were originated from both pulmonary and extrapulmonary TB patients, with different drug susceptibility profiles (pansusceptible, polyresistant and MDR) (Table 2). Among the 75 isolates, molecular cluster analysis based on 24-locus MIRU-VNTR genotypes identified 69 distinct genotypes, only including 5 clusters comprising from 2 (n = 4) to 3 isolates (n = 1) each (total of 11 isolates) and 64 unique types (Fig 1 and Table 2).

Out of these unique types, 5 could not be assigned into well-determined genotypic lineage in the MIRU-VNTR*plus* database, SpolDB4 and SITVIT database and as such were labeled as “unknown”. Importantly, the largest spoligotyping defined clusters were very efficiently subdivided by MIRUs, as e.g spoligotyping cluster ST42 (LAM), which formed a block of 29 isolates, was almost completely resolved, with only four remaining clustered isolates. Likewise, the second largest spoligotyping defined cluster ST50 (Haarlem) was also almost completely resolved, with only a single cluster of 2 isolates remaining. Thus, among the 75 isolates, the MIRU- based clustering rate corresponded 14.66% versus 76% for spoligotyping. The isolates within the five 24-locus MIRU-VNTR-based clusters showed identical spoligotype profiles, except in two cases, including a generic ST53/T1 and a ST42/LAM9 spoligotypes, and perhaps more unexpectedly, a ST50/Haarlem and a ST42/LAM9 spoligotypes, respectively.

When analyzed at the scale of the full isolate subset, groupings based on 24-locus MIRU-VNTR and spoligotyping results were fairly congruent (Fig 1 and Table 3). Two main strain groups were identified on the basis of the MIRU-VNTR-based tree, essentially composed of isolates with Haarlem spoligotypes (and isolates with generic T spoligotypes), and LAM spoligotypes (also with isolates with generic T spoligotypes), respectively. A few outliers with apparently discordant grouping were detected in either part of the tree, e.g., two ST42/LAM9 isolates (including one clustered with a T1 isolate) found among otherwise Haarlem branches.

**Table 3:** Detailed Results obtained including demoraphic, drug-resistance and genotyping information on 5 clusters including 11 isolates defined by 24-loci MIRU patterns from 75 *M*.*tuberculosis* strain isolated in Morocco.

ID	Year of Isolation	Sex	Age	City	Site of Infection	Spoligotype Description	Cluster(SIT/Clade)	MIRU24 profil	Cluster/MLVA MtbC15-9
17	2011	M	19	Rabat	Pulmonary	▪▪▪▪▪▪▪▪▪▪▪▪▪▪▪▪▪▪▪▪▪▪▪▪▪▪▪▪▪▪▫▪▫▫▫▫▪▪▪▪▪▪▪	Cluster A(50/H3)	233235322634425154233432	Cluster A1(9927–415)
79	2012	M	31	Rabat	Pulmonary	▪▪▪▪▪▪▪▪▪▪▪▪▪▪▪▪▪▪▪▪▪▪▪▪▪▪▪▪▪▪▫▪▫▫▫▫▪▪▪▪▪▪▪	Cluster A(50/H3)	233235322634425154233432	Cluster A1(9927–415)
26	2011	M	20	Sale	Extrapulmonary	▪▪▪▪▪▪▪▪▪▪▪▪▪▪▪▪▪▪▪▪▫▫▫▫▪▪▪▪▪▪▪▪▫▫▫▫▪▪▪▪▪▪▪	NC (ST42/LAM9)	134243352324126153332932	Cluster B1(358–51)
16	2011	M	31	Rabat	Extrapulmonary	▪▪▪▪▪▪▪▪▪▪▪▪▪▪▪▪▪▪▪▪▪▪▪▪▪▪▪▪▪▪▫▪▫▫▫▫▪▪▪▪▪▪▪	NC (ST50/H3)	1342243352324126153332932	Cluster B1(358–51)
51	2011	F	36	Oujda	Pulmonary	▪▪▪▪▪▪▪▪▪▪▪▪▪▪▪▪▪▪▪▪▪▪▪▪▪▪▪▪▪▪▪▪▫▫▫▫▪▪▪▪▪▪▪	NC (ST53/T1)	2432443323344251533431032	Cluster C1(Unk-15)
58	2011	M	24	Sale	Pulmonary	▪▪▪▪▪▪▪▪▪▪▪▪▪▪▪▪▪▪▪▪▫▫▫▫▪▪▪▪▪▪▪▪▫▫▫▫▪▪▪▪▪▪▪	NC (ST42/LAM9)	2432443323344251533431032	
166	2012	F	26	Meknes	Pulmonary	▪▪▪▪▪▪▪▪▪▪▪▪▪▪▪▪▪▪▪▪▫▫▫▫▪▪▪▪▪▪▪▪▫▫▫▫▪▪▪▪▪▪▪	Cluster B(ST42/LAM9)	244244321322225172346522	ClusterD1(10248-Unk)
168	2012	F	20	Meknes	Pulmonary	▪▪▪▪▪▪▪▪▪▪▪▪▪▪▪▪▪▪▪▪▫▫▫▫▪▪▪▪▪▪▪▪▫▫▫▫▪▪▪▪▪▪▪	Cluster B(ST42/LAM9)	244244321322225172346522	ClusterD1(10248-Unk)
169	2012	M	19	Sidi Kacem	Pulmonary	▪▪▪▪▪▪▪▪▪▪▪▪▪▪▪▪▪▪▪▪▫▫▫▫▪▪▪▪▪▪▪▪▫▫▫▫▪▪▪▪▪▪▪	Cluster B(ST42/LAM9)	244244321322225172346522	ClusterD1(10248-Unk)
22	2011	M	40	Fes	Pulmonary	▪▪▪▪▪▪▪▪▪▪▪▪▪▪▪▪▪▪▪▪▪▪▪▪▪▪▪▪▪▪▪▪▫▫▫▫▪▪▪▪▪▪▪	Cluster C(ST53/T1)	223234332334425153333712	Cluster E1(13387–15)
23	2011	F	31	Fes	Pulmonary	▪▪▪▪▪▪▪▪▪▪▪▪▪▪▪▪▪▪▪▪▪▪▪▪▪▪▪▪▪▪▪▪▫▫▫▫▪▪▪▪▪▪▪	Cluster C(ST53/T1)	223234332334425153333712	Cluster E1(13387–15)

ID: Identifying number

MLVA-MtbC15-9: Multi Locus Variant Allele-Mycobacterium tuberculosis complex15-9

Regarding the allelic diversity of the MIRU-VNTR loci, the discriminatory power was calculated using HGDI (summarized in Table 4). The allelic diversity of the loci was classified as very discriminant [Hunter-Gaston Index (HGI)>0.6], moderately discriminant (0.3<HGI<0.6) and poorly discriminant (HGI<0.3) [33]. Among the 24-loci, we note that loci MIRU 4052, 802, 2996, 2163b, 3690, 1955, 424, 2531, 2401 and 960 were highly discriminative (HGDI>0.6) whereas loci MIRU 577, 4156, 2165, 2347, 3192 and 154 were moderately discriminative (0.6<HGDI<0.3) and loci MIRU 2059, 3007, 1644, 580, 3171, 2461, 4348, 2687 were poorly discriminative (HGDI<0.3).

**Table 4:** Allelic polymorphism of 24 mycobacterial interspersed repetitive units (MIRUs) loci from 75 MTB isolates from patients with tuberculosis in different regions of Morocco

MIRU- VNTR Locus	Allele number													Allelic diversity (h)	Conclusion
	0	0s	1	2	3	4	5	6	7	8	9	10	11		
**154**			18	57										0.36	Moderately discriminant
**424**			3	31	17	23		1						0.68	Very discriminant
**577**				6	27	40	2							0.57	Moderately discriminant
**580**		1	3	70	1									0.12	Poorly discriminant
**802**	1		9	9	23	24	4	3	1	1				0.77	Very discriminant
**960**				5	10	42	16	2						0.61	Very discriminant
**1644**			2	6	65	2								0.23	Poorly discriminant
**1955**			1	28	29	1	5	1	10					0.68	Very discriminant
**2059**			14	61										0.29	Moderately discriminant
**2163b**			2	19	33	9	4	7		1				0.71	Very discriminant
**2165**				41	31	3								0.52	Moderately discriminant
**2347**				25	1	49								0.45	Moderately discriminant
**2401**			17	27		31								0.64	Very discriminant
**2461**			1	72		2								0.07	Poorly discriminant
**2531**				2	10	4	33	25	1					0.67	Very discriminant
**2687**			75											-0.01	Poorly discriminant
**2996**			2	4	8	7	32	10	8	2	2			0.76	Very discriminant
**3007**			1	8	62	4								0.27	Moderately discriminant
**3171**			1	3	71									0.09	Poorly discriminant
**3192**				1	52	21	1							0.43	Moderately discriminant
**3690**			1	19	35	4	7	7	1				1	0.69	Very discriminant
**4052**	1		4	7	4	5	15	13	16	5	2	3		0.86	Very discriminant
**4156**			3	37	35									0.53	Moderately discriminant
**4348**				74	1									0.01	Poorly discriminant

## Discussion

This is the first study to explore the usefulness of standard 24-locus based MIRU-VNTR typing for molecular epidemiological study of MTBC strains from Morocco. Hitherto, MTBC population structure and tuberculosis transmission were only studied at the national level by using spoligotyping alone or in conjunction with 12-loci MIRUs [16,17]. In comparison to these two typing methods, the discriminatory power of 24-locus MIRU-VNTR typing has been shown to be higher in a number of other settings in Europe and elsewhere [4,34,35], and often similar to that of IS*6110*-based RFLP when comparison with the latter method were made [6–9].

In order to explore the informative value of 24-locus MIRU-VNTR typing, we selected 75 representatives from a baseline sample of 168 MTBC isolates screened by spoligotyping from 10 cities with high burden of TB, for which we also determined the drug resistance profiles. As summarized in Table 2, 24-locus based MIRU-VNTR typing largely reduced the clustering defined by spoligotyping alone or even by spoligotyping combined with 12-locus MIRU-VNTR typing, from 76% and 48% to 14.6%, respectively. It is noteworthy that this effect was particularly noticed for large clusters initially defined by spoligotypes such as ST42 (LAM) and ST50 (Haarlerm), which are predominant in Morocco, as seen in this study as well in previous reports [16,17]. After analysis with 24 loci, these initial spoligotype-based clusters were reduced to a few clusters of at most three isolates. Hence, no correlation was apparent between particular MIRU-VNTR types and drug resistance profile, except for three MDR isolates that were confirmed to be part of a same familial outbreak (see just below). Contact tracing could not be conducted for all these remaining clustered isolates in order to confirm TB transmission assumed from molecular clustering. It is noteworthy however that the single cluster of three isolates corresponded to two previously treated MDR-TB cases and one new MDR-TB case known to be contact a same index case and belonging to a same family, albeit residing in 3 different administrative regions (Fes-Boulmane, Meknes-Tafilalet and Garb-Chrarda-BniHssen) during 2011. The consistent clustering of these three cases lends some degree of confidence to the overall reliability of the molecular results obtained. This is also suggested by the overall degree of congruence observed between groupings on the basis on the 24-locus MIRU-VNTR-based tree and grouping of the corresponding spoligotypes. Only a few outliers were detected, for which samples were unavailable for repeat experiments in order to see if these few discordant groupings reflected some homoplasy linked to spoligotyping or MIRU-VNTR data [36], or technical errors. In this sample collection, two different isolates were also obtained from one patient with both pulmonary and extrapulmonary TB. Both spoligotyping and 24-locus MIRU-VNTR typing showed that the strain isolated from sputum defined as ST273/LAM9 and MLVA MTBC Unk-488 differed from the one isolated from pleural liquid clinical sample, defined as ST784/T2 and MLVA MTBC Unk-85. This concordant result obtained by independent typing methods suggests a case of mixed infection with compartmentalization of different strains in distinct body sites [37]. Considering the full set of spoligotyping data obtained for the 168 study isolates, our results confirm that MTBC isolates in our country are essentially limited to evolutionary modern principle genetic group (PGG)2/3 strains (namely LAM, Haarlem, and T), as found in previous reports and different patient populations [16,17]. Overall, when our results are compared with those of these previous reports, it seems that MTBC population structure in Morocco is highly stable, with almost same spoligotype distributions from 2002 to 2012, and highly homogeneous. Strikingly, each of the 3 predominant LAM, Haarlem and T families was characterized by one or two predominant SITs (i.e.SIT42 for LAM, SIT50 for Haarlem and SIT53 for T), almost always representing the prototype of each spoligotype family with large geographical distribution described in different databases [21,22]. It can be speculated that this predominance of a few SITs, especially that of LAM family, reflects some founder effect linked to the introduction of the corresponding MTBC clonal branches in the region. In contrast, although we found a total of 49 profiles identified among the 168 isolates, we did not find spoligotypes with strong local phylogeographical specificity (Table 2). We only identified 5 Beijing isolates from three different cities (Marrakech, Rabat and Casablanca), while a previous study also detected a few isolates with such genotype in Marrakech, Fes and Sale [16]. This low prevalence of Beijing strains in Morocco plausibly reflects the low level of human immigration from East Asia, where this strain lineage largely prevails and probably originally emerged according to recent analyses [11]. MTBC isolates with a Beijing genotype are often, albeit not always associated with MDR-TB, especially Eurasia and East-Asian countries [11,38–41]. In our study, a single Beijing isolate obtained from an extrapulmonary TB patient in Casablanca, was MDR while the 4 other isolates were pansusceptible. This is in accordance with previous studies, suggesting little to no association of Beijing genotypes with MDR-TB in the country [12,16]. However, more vigorous investigations are needed to further study this question and to trace the possible origins of the Beijing isolates identified in our and previous studies [16]. We also detected 4 isolates with another, more geographically specific spoligotype, belonging to the SIT61/CAM spoligotype family in 3 cities: Fes (n = 1), Marrakech (n = 1) and Rabat (n = 2). As this spoligotype has strong phylogeographical specificity to Cameroon and its neighboring countries in West Africa [21,22,42], the presence of these strains in Morocco is suggestive of importation by sub-Saharan migrants to and across Morocco [43–45]. Finally, we detected two isolates of T2 spoligotype family, and one with a SIT443/U pattern, none of which were previously described in Morocco. Such patterns have been essentially reported from different region of Asia and Canada [21], and Asia USA and Europe, respectively [21].

Also in line with finding from other, previously studied Moroccan patient population [16,17], we observed very little evidence of multidrug resistance with a frequency of only 3.0% among the new cases in this study. Among the remaining cases, 85.7% were pansusceptible, and the remaining 10.1% showed resistance to one or more drugs.

In conclusion, although this study was limited by a relatively small sample size, the obtained results support the use of 24-locus MIRU-VNTR typing in our country, for substantially reducing the degree of over estimation of epidemiological links inferred among isolates analyzed by spoligotyping alone or in combination with 12-locus MIRU-VNTR typing. This use should be especially beneficial to distinguish the many strains that apparently share a few highly predominant prototypic spoligotypes. We therefore hope that its implementation will help enhanced TB control program in Morocco to reduce the TB burden in the country [4,30,36]. As a future cost-effective strategy and when possible, whole genome sequencing (WGS) could then be specifically applied on the remaining MIRU-VNTR-based clusters to further test their possible significance in terms of ongoing TB transmission, given the superior resolution power provided by WGS to resolve TB outbreaks [46,47].

## Supporting Information

S1 Table Description of 39 different spoligotype patterns obtained from the 168 *M*.*tuberculosis* clinical isolates in Morocco according to the SITVIT database. (PDF)
