# Supplementary material for: Molecular Typing of Mycobacterium Tuberculosis Complex by 24-Locus Based MIRU-VNTR Typing in Conjunction with Spoligotyping to Assess Genetic Diversity of Strains Circulating in Morocco
Source: PLoS One. 2015 Aug 18;10(8):e0135695. doi: 10.1371/journal.pone.0135695 (PMC4540494; doi:10.1371/journal.pone.0135695)
Supplement: S1 Table — (PDF) [file pone.0135695.s001.pdf]

SIT (Clade) Octal Number & Spoligotype Description	n (%) in study
1 (Beijing) 000000000003771	5(2.97)
33 (LAM3) 776177607760771	4(2.38)
34 (S Clade) 776377777760771	2(1.19)
42 (LAM9) 777777607760771	49(29.16)
47 (H1) 777777774020771	7(4.16)
50 (H3) 777777777720771	17(10.11)
53 (T1) 777777777760771	19(11.30)
61 (CAM) 777777743760771	4(2.38)
62 (H1) 777777774020731	3(1.78)
64 (LAM6) 777777607560771	1(0.59)
93 (LAM5) 777737607760771	1(0.59)
205 (T1) 737777777760771	1(0.59)
252 (LAM9) 777477607760771	1(0.59)
268 (H3) 777777677720771	1(0.59)
273 (LAM9) 777617607760771	1(0.59)
291 (T1) 777777677760771	1(0.59)
388 (LAM9) 737777607760771	1(0.59)
390 (H3) 777777777620771	1(0.59)
433 (H3) 757777777320771	2(1.19)
443 (U Clade) 57777777320771	1(0.59)
462 (T1) 777777777560771	1(0.59)
602 (T1) 777777770000771	1(0.59)

615 (H3) 777777777720770	1(0.59)
731 (LAM9) 777761607760771	2(1.19)
741 (H3) 777777757720771	4(2.38)
746 (H3) 777777777520771	1(0.59)
784 (T2-S) 776377777760731	2(1.19)
822 (LAM9) 757777607760771	2(1.19)
964 (LAM9) 437777607760771	1(0.59)
1070 (U Clade) 777777607760371	3(1.78)
1071 (LAM9) 777771607760771	3(1.78)
1072 (LAM9) 777731607760771	1(0.59)
1074 (LAM9) 777777607760761	5(2.97)
1111 (T1) 377777777760760	1(0.59)
1537 (LAM3) 776137607760731	4(2.38)
1557 (H1) 777677774020771	1(0.59)
1708 (LAM9) 776777607760771	1(0.59)
1743 (H3) 767777777720631	1(0.59)
1891 (H3) 777777773720771	1(0.59)
